# Identification of active compounds in *Vernonia anthelmintica* (L.) willd by targeted metabolome MRM and kaempferol promotes HaCaT cell proliferation and reduces oxidative stress

**DOI:** 10.3389/fphar.2024.1343306

**Published:** 2024-04-09

**Authors:** Wen Hu, Hongjuan Wang, Kaixiao Li, Zixian Lei, Fang Xiang, Jun Li, Xiaojing Kang

**Affiliations:** ^1^ Department of Dermatology and Venereology, People’s Hospital of Xinjiang Uygur Autonomous Region, Urumqi, China; ^2^ Xinjiang Clinical Research Center for Dermatologic Diseases, Urumqi, China; ^3^ Xinjiang Key Laboratory of Dermatology Research (XJYS1707), Urumqi, China

**Keywords:** vitiligo, Vernonia anthelmintica, kaempferol, PI3K/AKT signaling, HaCaT cell

## Abstract

**Introduction:** *Vernonia anthelmintica* (L.) Willd. is a traditional treatment for vitiligo in Xinjiang. However, its therapeutic mechanism remains unclear owing to its complex composition and limited research on its chemical profile.

**Methods:** We employed a targeted metabolome approach, combining selective reaction monitoring/multiple response monitoring (SRM/MRM) with high-performance liquid chromatography and MRM mass spectrometry to quantitatively analyze the flavonoid constituents of *Vernonia anthelmintica*. We also used network pharmacology and molecular docking to identify potential vitiligo-linked compounds and targets of *V. anthelmintica* seeds. Additionally, we assessed HaCaT cell proliferation by AAPH-induced**,** alongside changes in SOD activity and MDA content, following treatment with *V. anthelmintica* components. Finally, flow cytometry was used to detect apoptosis and ROS levels.

**Results and Discussion:** We identified 36 flavonoid compounds in *V. anthelmintica* seeds, with 14 compounds exhibiting druggability. *AKT1*, *VEGFA*, *ESR1*, *PTGS2*, and *IL2* have been identified as key therapeutic target genes, with PI3K/AKT signaling being an important pathway. Notably, kaempferol, one of the identified compounds, exhibited high expression in network pharmacology analysis. Kaempferol exhibited a strong binding affinity to important targets. Further, kaempferol enhanced HaCaT cell viability, inhibited apoptosis, reduced MDA levels, suppressed ROS activity, and upregulated SOD activity, increase the expression of cellular antioxidant genes, including HO-1, GCLC, GCLM, Nrf2, NQO1 and Keap1, providing significant protection against oxidative stress damage *in vitro*. Here, we present the first comprehensive study integrating SRM/MRM approaches and network analysis to identify active flavonoid compounds within *V. anthelmintica* (L.) Willd. Moreover, we revealed that its active ingredient, kaempferol, offers protection against AAPH-induced damage in keratinocytes, highlighting its potential as a clinical resource.

## 1 Introduction

Vitiligo stands as the most common skin depigmentation disease, characterized by chronic depigmentation and milk-white lesions on the skin, with a global prevalence of 0.5%–2% (Krüger and Schallreuter, 2012). Vitiligo can have a detrimental impact on appearance, affect social interaction, work experiences, marriage prospects, thereby resulting in social discrimination, which can promote the development of anxiety, depression, and other mental health disorders (Dabas et al., 2020; Wang et al., 2022). Clinically, vitiligo often co-occurs with hyperthyroidism, diabetes, alopecia areata, and other autoimmune disorders and has the capacity to induce the onset of psoriasis, malignant tumors, bronchial asthma, and other diseases (Amer and Gao, 2016; Zhang et al., 2022).

Oxidative stress damage is pivotal in the etiology of vitiligo, being considered as one of the most crucial initiators of this condition (Di Dalmazi et al., 2016; Chen et al., 2021). The secretion of growth factors, release of pro-inflammatory cytokines, and activation of inflammasomes within keratinocytes can exacerbate oxidative stress and promote immune responses, thus inducing or exacerbating the development of vitiligo (Chen et al., 2019). Therefore, antioxidant therapy has emerged as a promising strategy for the treatment of vitiligo.

*Vernonia anthelmintica* (L.) Willd. is an annual herb of the Compositae family. Notably, its habitat is exclusive to Kashgar, Aksu, and other areas in Xinjiang and India (Zhou et al., 2012; Hu et al., 2023). However, the chemical composition of *Vernonia anthelmintica* (L.) Willd. is complex and remains relatively unexplored worldwide. Previous studies have shown that the most effective ingredients for treating of vitiligo were flavonoids (Orhan and Khan, 2014; Liu-Smith and Meyskens, 2016). However, current investigations into *V. anthelmintica* (L.) Willd., have predominantly centered on total flavonoid extraction, with few studies focusing on individual flavonoid monomers and their respective therapeutic targets. Recent studies have suggested that kaempferol may be the primary active component of *V. anthelmintica* (L.) Willd. (Liu-Smith and Meyskens, 2016). Kaempferol is a flavonoid, commonly found in various Chinese plants, including tea, legumes, and plant-derived foods. Kaempferol has been demonstrated to play an important role in inflammatory diseases such as rheumatoid arthritis, systemic lupus erythematosus, and ankylosing spondylitis (Du et al., 2018; Pan et al., 2018; Wang et al., 2019a). However, the precise molecular mechanisms underlying the role of kaempferol in treating vitiligo remain unclear.

The targeted metabolome selective reaction/multiple response monitoring (SRM/MRM) approach enabling the detection and analysis of specific metabolite groups, yields absolute quantitative results for target metabolites. This method holds significant value, particularly in elucidating the complex compositions of traditional Chinese medicine (Awasthi et al., 2018). Network pharmacology is a technique that establishes associations between drugs and diseases by constructing intricate networks, offering insights into the potential mechanism of action of these medications (Wang et al., 2021; Ko et al., 2022; Noor et al., 2022; Liu et al., 2023). The integration of network pharmacology and molecular docking analyses emerges as an important approach for exploring key compounds and targets intricate relationships.

Therefore, here, we employed the SRM/MRM method to isolate 36 flavonoid compounds from *V. anthelmintica* (L.) Willd. Subsequently, we conducted a systematic network analysis to investigate the relationships between herbs, bioactive compounds, and putative target genes associated with *V. anthelmintica* (L.) Willd. Compounds that exhibited druggability were then screened for oral bioavailability (OB) and drug-likeness (DL). Putative target genes of these compounds and vitiligo-related genes were subsequently retrieved from public databases. By constructing a network that links the compounds with druggability derived from *V. anthelmintica* (L.) Willd. with their respective target genes, we aimed to identify key compounds and potential targets implicated in the treatment of vitiligo. Overall, this study demonstrated that kaempferol holds significant potential as a novel treatment strategy for vitiligo.

## 2 Materials and methods

### 2.1 Chemicals and reagents

Kaempferol, AAPH, superoxide dismutase (SOD), malondialdehyde (MDA), and cytotoxicity assay (CCK-8) kits were obtained from Solarbio (Beijing, China). 3,5-dimethylphenol was obtained from Life Technologies (Carlsbad, CA, United States). Phosphate-buffered saline (PBS) was procured from Biological Industries (Ridgefield, CT, United States). Penicillin–streptomycin solution was obtained from ScienCell (San Diego, CA, United States). Dulbecco’s modified Eagle’s medium (DMEM) and trypticase were purchased from HyClone (Logan, UT, United States). Fetal bovine serum (FBS) was procured from Gibco (Waltham, MA, United States). A BCA Protein Quantitation Kit was obtained from Thermo Fisher Scientific (Waltham, MA, United States).

### 2.2 V. anthelmintica (L.) Willd. extract preparation

*Vernonia anthelmintica* (L.) Willd. was procured from the Eric Uygur medical room in Urumqi (Xinjiang Province, China). Subsequently, the chief pharmacist of the Pharmaceutical Research Institute of the Xinjiang Uygur Autonomous Region identified this sample as dried mature seeds of *V. anthelmintica* (L.) Willd. The extraction process involved placing the seed powder in a sealed contained and adding 50 mL of 60% ethanol solution. This mixture was then subjected to ultrasonic extraction at 40°C for 50 min, followed by filtration. Subsequently, 40 mL of 60% ethanol solution was added to the residue before further ultrasonic extraction at 40°C for 20 min, followed by filtration. The filtrates from both steps were then combined, before a final filtration with 10 mL of hot ethanol solution (60% volume fraction, 56°C preheat). The resulting filtrate was then cooled down to 25°C and a 60% ethanol solution was adjusted to 100 mL. Finally, the sample was shaken and left to stand for future use.

### 2.3 Isolation, characterization, and quantification of total flavonoids

First, we analyzed the *V. anthelmintica* (L.) Willd. extract using SRM/MRM. Initially, 500 µL of the sample was placed in a 2-mL centrifuge tube, for centrifugation at 4000 rpm for 5 min. The supernatant was then removed, with 5 µL aliquots being placed into a 96 well plate for mass analysis. The sample was then placed in an ACQUITY UPLC I-Class system (Waters, American), for SRM/MRM under the following conditions: An autosampler temperature of 4°C; Mobile phase A and B of 0.1% formic acid in water and acetonitrile, respectively. The gradient elution procedure used was as follows: B increased from 5% to 20% between 0 and 3 min; B was then maintained at 20% between 3 and 4.3 min; at 4.3–9 min B increased from 20% to 45%; then, B increased from 45% to 98% between 9 and 11 min; at 11–13 min B was maintained at 98%; B then decreased from 98% to 5% between 13 and 13.1 min; and, finally, B was maintained at 5% at 13.1–15 min. The flow rate of this system was set at 400 μL/min, with an injection volume of 5 µL.

For mass spectrometry (MS), a Sciex 5500 QTRAP, mass spectrometer (AB SCIEX, American) was used, which was capable of operating in both positive and negative ion switching modes. The positive ion source parameters were as follows: source temperature, 550°C; Gas 1, 55 psi; Gas 2, 50 psi; curtain gas (CRU), 30 psi; ion spray voltage floating (ISVF), 5–500 V. The negative ion source parameters were as follows: source temperature, 550°C; Gas 1, 55 psi; Gas 2, 50 psi; CRU, 30 psi; ISVF, 4500 V.

### 2.4 Network pharmacology predictive analysis

#### 2.4.1 Identifying and analyzing target components and vitiligo

In this study, active ingredients of *V. anthelmintica* (L.) Willd. were screened using the Traditional Chinese Medicine Systems Pharmacology database (https://tcmsp-e.com/tcmsp.php, accessed 20 March 2023). Oral bioavailability (OB) and drug-likeness (DL) were considered the most important pharmacokinetic parameters for absorption, distribution, metabolism, and excretion (ADME). Therefore, these active ingredients were screened using the following filter criteria: OB ≥ 30% and DL ≥ 0.18. Complete compound information was then collected, including the Mol ID, structure, and relevant target names. Meanwhile, disease-related targets were retrieved using the DisGeNET platform (https://www.disgenet.org/, accessed on 21 March 2023), which identified gene–disease associations liked to the term “vitiligo.” The targets were normalized using the UniProt database (https://www.uniprot.org/, accessed on 21 March 2023), with “human species” and “reviewed” filter criteria. To identify common targets of active ingredients and vitiligo, the screened active ingredients targets and vitiligo targets were visualized in a Venn diagram (http://bioinformatics.psb.ugent.be/webtools/Venn/, accessed on 29 March 2023).

#### 2.4.2 PPI network construction and key target analysis

To explore the interactions between the targets of active ingredients that treat vitiligo, we imported the common targets of *V. anthelmintica* (L.) Willd. active ingredients and vitiligo into an online platform, STRING (version 11.0.6, https://cn.string-db.org/, accessed on 29 March 2023), which was used to construct a PPI network. The PPI network results were then analyzed and visualized using Cytoscape v.3.9.1. Further, a Cytoscape plug-in, Centiscape 2.2, was used to identify high-interaction targets based on the closeness unDir, betweenness unDir, and degree unDir thresholds.

#### 2.4.3 GO and KEGG enrichment analysis

GO and KEGG pathway enrichment analyses were conducted using the Metascape platform (http://metascape.org/gp/index.html#/main/step1, accessed 29 March 2023). The primary GO terms associated with potential targets were analyzed, identifying biological process (BP), cellular component (CC), and molecular function (MF) enrichment. KEGG pathways analysis was then performed to identify enriched pathways. The significant enrichment terms (*p* ≤ 0.01) were then visualized using an online platform (http://www.bioinformatics.com.cn/, accessed on 29 March 2023).

#### 2.4.4 Component–target protein molecular docking

The 2D structures of the identified active ingredients were downloaded from PubChem (https://pubchem.ncbi.nlm.nih.gov/, accessed on 30 March 2023), converted into 3D structures using Chem3D software, and saved in a docking ligand mol2 format. Alternatively, the 3D structures of the previously identified key proteins were obtained from the PSCB PDB platform (https://www.rcsb.org/, accessed 30 March 2023). The key proteins were removed from the organic solvents associated with them before and adding hydrogen using PyMOL software. AutoDock software (v. 4.2.6) was then used for molecular docking simulation. Finally, the molecular docking results were visualized using PyMOL software.

### 2.5 *In vitro* cell culture and treatment

In this study, human keratinocytes (HaCaT; Kunming Cell Bank, Chinese Academy of Sciences, China) were cultured in DMEM supplemented with 10% FBS and a 1% penicillin–streptomycin solution. These cells cultured grown at 37°C in a humidified atmosphere with 5% CO_2_ and 10% a copper sulfate solution. Subsequent experiments were performed 24 h after the cells were seeded. Specifically, HaCaT cells were seeded at a density of 8×10^4^ cells/well into 96-well plates for 12 h and treated with various concentrations (10, 15, 20, 25, 30, 40, 50, or 60 mM) of AAPH. Alternatively, a subset of HaCaT cells was pre-treated with various concentrations (10, 20, 30, 40, 60, 80, or 100 µM) of kaempferol within the medium for 12 h to determine the optimal treatment concentration. Subsequently, the pre-treated cells were cultured for an additional 12 h with 25 mM AAPH to induce oxidative damage. Three replicates were conducted per group for all the experiments; untreated cells and cells treated solely with AAPH were used as control groups.

### 2.6 Cell viability assay

To evaluate the cellular toxicity of AAPH and the protective effects of kaempferol, we employed the CCK-8 assay on HaCaT cells. After treatment with the aforementioned concentrations of AAPH and kaempferol, 10 µL of the CCK-8 kit solution was added to each well. Subsequently, the cells were incubated for 2 h at 37°C. The absorbance of each sample was measured using a microplate reader (Thermo, Multiskanm FC, American).

### 2.7 Biochemical analysis

Cold PBS was used to collect kaempferol-treated and untreated HaCaT cell samples following centrifugation at 157 *g* for 3 min, according to the instructions provided in the respective enzyme kits. These cells were then homogenized with cold PBS, and corresponding commercial kits were used to extract the supernatant; this supernatant was then analyzed for MDA and SOD activity levels.

### 2.8 Apoptosis assay

To assess the protective effect of kaempferol against oxidative stress–induced apoptosis in HaCaT cells, we employed a Hoechst assay. Cells pre-treated with 10, 20, and 30 µM kaempferol were used as the experimental groups for this assay. After cell treatment, 5 μg/mL Hoechst staining solution was added to the samples, before incubation for 10–15 min at 25°C in the dark. Subsequently, 400 µL of 1× binding buffer was added to the samples, ensuring thorough mixing before being placed on ice. Finally, cellular images were taken within 1 h under an Olympus fluorescence microscope. This experiment was repeated thrice.

### 2.9 SOD activity

In this study, SOD activity in HaCaT cells was evaluated using the tetrazolium nitro blue method. First, HaCaT cells in the logarithmic growth stage were prepared by digestion and PBS washing. The cell suspensions were then centrifuged at 157 *g* for 5 min. HaCaT cells were seeded in 6-well plates at a density of 1×10^6^ cells/well, and cultured for 12 h at 37°C in a humidified atmosphere with 5% CO_2_. When the cells had adhered to the wells and reached a density of approximately 80%, they were treated with various concentrations (10, 20, or 30 µM) of kaempferol for 12 h. Subsequently, the pre-treated cells were cultured for an additional 12 h with 25 mM AAPH. The cell suspensions were then mixed thoroughly using a vortex mixer (ice bath; power, 20%/200 W; ultrasound, 3-s, 10-s intervals, 30 repeats) before centrifugation at 8000 *g* at 4°C for 10 min. Subsequently, the corresponding reagents were added to the cell suspension according to the manufacturer’s instructions. Finally, the mixture was thoroughly mixed before culturing at 37°C for 30 min. Cell suspension samples were then placed in a 1-mL glass cuvette, and maximal absorbance was measured at 560 nm using a spectrophotometer (Thermo, Multiskanm FC, American). One unit of SOD activity was defined as the amount of enzyme required for 50% activity inhibition.

### 2.10 MDA assay

In this study, MDA concentration was used to assess lipid peroxidation in the HaCaT cells. First, the cell suspensions were mixed thoroughly using a vortex mixer (ice bath; power, 20%/200 W; ultrasound 3-s, 10-s intervals, 30 repeats) before centrifugation at 8000 *g* at 4°C for 10 min. The corresponding reagents were then added to the cell suspensions according to the manufacturer’s instructions. Subsequently, the mixture was thoroughly mixed and incubated at 100°C for 60 min, cooled in an ice bath, and centrifuged at 10,000 ×*g* for 10 min. Finally, 200 µL of each supernatant was transferred into a microglass cuvette or 96-well plate; maximal absorbance was then determined at 450 nm, 532 nm, and 600 nm using a spectrophotometer. MDA content was calculated according to the identified protein concentration.

### 2.11 Statistical analysis

All statistical analyses were conducted using SPSS (version 22.0; IBM Corp., Armonk, NY, United States). Statistical significance was set at *p* < 0.05.

## 3 Results

### 3.1 Identifying and sorting flavonoids compounds in V. anthelmintica (L.) Willd

**FIGURE 1:**
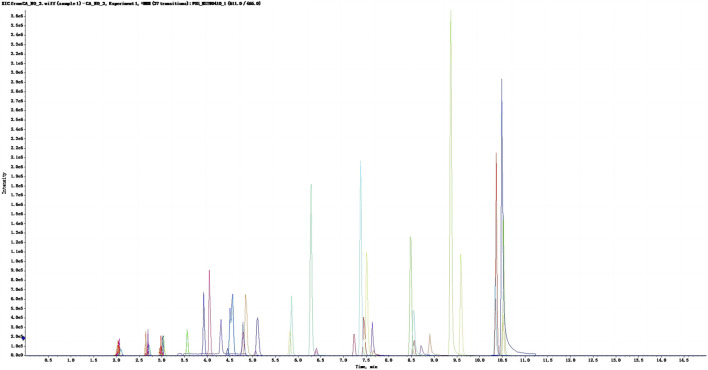
XIC profile of the standard mixture. The abscissa represents the time (min), the vertical coordinates represents ionic intensity.

Ethanolic extracts obtained from *V. anthelmintica* (L.) Willd. Seeds were subjected to quantitative flavonoid testing, which revealed a total flavonoid content of 0.97 g per 100 g. Then, we conducted SRM MS analysis following high-performance liquid chromatography and SRM/MRM; this analysis identified 36 flavonoid compounds. These compounds were subsequently categorized according to their structure: flavonoid glycosides (9/36), isoflavonoids (9/36), flavans (7/36), flavones (5/36), and others (6/36). Notably, all of the resultant curves exhibited excellent linear regressions with coefficients of determination (*R* ^2^) ranging from 0.9963 to 0.9999 (Figure 1). The top five compounds with the highest contents were saccarol (12650.98 ng/100 g), isquercitrin (7293.86 ng/100 g), meltein (4503.36 ng/100 g), violet (4320.83 ng/100 g), and naringenin (3390.59 ng/100 g).

Overall, 36 flavonoid compounds were obtained from the ethanolic extract of *V. anthelmintica* (L.) Willd. Seeds. To identify the active ingredient of this herb, we utilized databases to screen bioactive compounds and ADME properties in *V. anthelmintica* (L.) Willd. When the screening criteria (OB ≥ 30%; DL ≥ 0.18) were applied, we identified 14 compounds with druggability in *V. anthelmintica* (L.) Willd. (Table 1).

**TABLE 1:** List of 14 compounds with druggability, identified using network pharmacology analysis of *Vernonia anthelmintica* (L.) Willd. seeds.

Molecule ID	Component	OB (%)	DL	MW	AlogP	Caco-2
MOL004564	Kaempferolm pferide	73.41	0.27	300.28	2.02	0.43
MOL005190	Eriodictyol	71.79	0.24	288.27	2.03	0.17
MOL002975	Butin	69.94	0.21	272.27	2.3	0.3
MOL000392	Formononetin	69.67	0.21	268.28	2.58	0.78
MOL004576	Taxifolin	57.84	0.27	304.27	1.49	0.23
MOL000492	catechin	54.83	0.24	290.29	1.92	−0.03
MOL000354	Isorhamnetin	49.6	0.31	316.28	1.76	0.31
MOL000073	Epicatechin	48.96	0.24	290.29	1.92	0.02
MOL000098	Quercetin	46.43	0.28	302.25	1.5	0.05
MOL000422	Kaempferol	41.88	0.24	286.25	1.77	0.26
MOL013428	Sakuranetin	41.24	0.72	594.62	−0.21	−1.59
MOL000006	Luteolin	36.16	0.25	286.25	2.07	0.19
MOL001792	Liquiritigenin	32.76	0.18	256.27	2.57	0.51
MOL002322	Isovitexin	31.29	0.72	432.41	−0.06	−1.24

### 3.2 Compound–target network analysis

**FIGURE 2:**
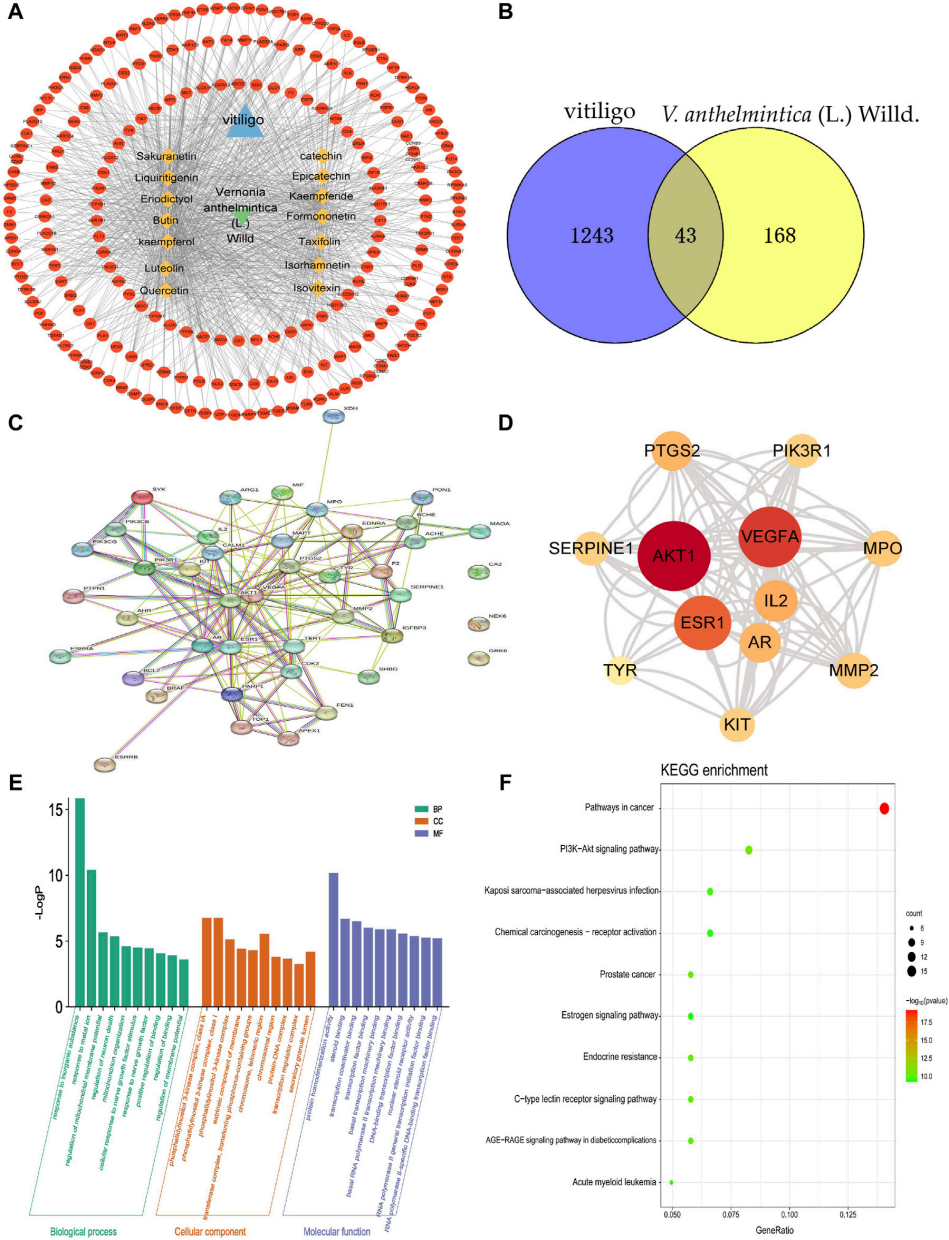
Primary active components of *Vernonia anthelmintica* (L.) Willd. and the prediction of biological targets involved in the treatment of vitiligo. **(A)** Network of interactions between active ingredients, action targets, and vitiligo. The circle represents the potential action targets of active ingredients; the rhombus represents the active ingredients of *V. anthelmintica* (L.) Willd.; and the triangle represents vitiligo. **(B)** Identification of 43 overlapping differentially expressed genes associated with the targets of vitiligo (blue) and the pharmaceutical components of *V. anthelmintica* (L.) Willd. (yellow). **(C)** Protein–protein interaction network diagram of *V. anthelmintica* (L.) Willd. intervention disease targets; the thicker the connected edges, the greater the interaction between the two nodes. **(D)** Top 10 genes associated with *V. anthelmintica* (L.). Willd. in the treatment of vitiligo; deeper shades of red indicate higher degree values. **(E)** GO enrichment analysis of the vitiligo-related disease targets associated with the primary active components of *Vernonia anthelmintica* (L.) Willd. **(F)** KEGG pathway enrichment analysis of the vitiligo-related disease targets associate with the primary active components of *V. anthelmintica* (L.) Willd.

In this study, we obtained a total of 1286 vitiligo-related genes from various databases, including GeneCards, OMIM, and TTD. Subsequently, we identified a total of 211 target genes associated with the 14 compounds selected for further analysis, eliminating any overlapping targets obtained from the GeneCards, PubChem, and Swiss target prediction databases. After identifying the compound-target vitiligo-related genes, we visualized the corresponding compound-target interaction network, constructing a network that consisted of 227 nodes and 963 edges (Figure 2A). The five compounds with the highest degree of interaction in this network were kaempferol, isorhamnetin, kaempferide, quercetin, and liquiritigenin (degree = 101). Further, by taking the intersection between compound–target genes and vitiligo-related genes from the Wayne database, we identified the compounds target and vitiligo-related gene set (Figure 2B); this consisted of 43 common targets, which were used for further analysis.

### 3.3 Construction of pharmaceutical component–disease targets and PPI networks

As shown in Figure 2C, the PPI network contained candidate targets of pharmaceutical ingredients for treating vitiligo and its interacting proteins. This network comprehensively summarizes the internal network of the pharmaceutical ingredients of *V. anthelmintica* (L.) Willd. involved in treating vitiligo. Finally, the NetworkAnalyzer and CytoNCA functions in Cytoscape were used to identify the core targets by analyzing various key properties, including the closeness, degree, and betweenness centrality (BC). In this analysis, a greater degree value indicates a more significant role in the network. r. The top five nodes with the highest degree values were determined to be *AKT1* (d = 60), *VEGF-A* (d = 50), *ESR1* (d = 44), *IL2* (d = 30), and *MPO* (d = 24). The degree values of the top 10 nodes are shown in Figure 2D. Therefore, these core targets are postulated to play a major role in the treatment of vitiligo using *V. anthelmintica* (L.) Willd.

### 3.4 GO enrichment and KEGG pathway analyses

GO enrichment analysis revealed that the biological processes associated with the targets of the active components in *V. anthelmintica* (L.) Willd. (Figure 2E), and vitiligo primarily involve responses to inorganic substances, regulation of mitochondrial membrane potential, and mitochondrial organization. The cell components identified were predominantly enriched in the phosphatidylinositol 3-kinase complex, the extrinsic component of the membrane, and the protein–DNA complex. The associated molecular functions primarily involved protein homodimerization, steroid binding, and transcription coactivator binding.

Biological pathway enrichment analysis of the target genes was also performed using the KEGG database. The enriched pathways identified by KEGG analysis included the PI3K−Akt signaling pathway, chemical carcinogenesis receptor activation, the estrogen signaling pathway, endocrine resistance, and C-type lectin receptor signaling pathways. Among them, the PI3K-AKT signaling pathway was determined to be particularly important. As shown in Figure 2F, the PI3K-AKT signaling pathway was linked to several key functions, including apoptosis, cell proliferation, cell cycle regulation, protein synthesis, and metabolism. Furthermore, it exhibits synergistic crosstalk with various signaling pathways, including the VEGF, MAPK, JAK/STAT, NFκB signaling pathways.

### 3.5 Molecular docking

**FIGURE 3:**
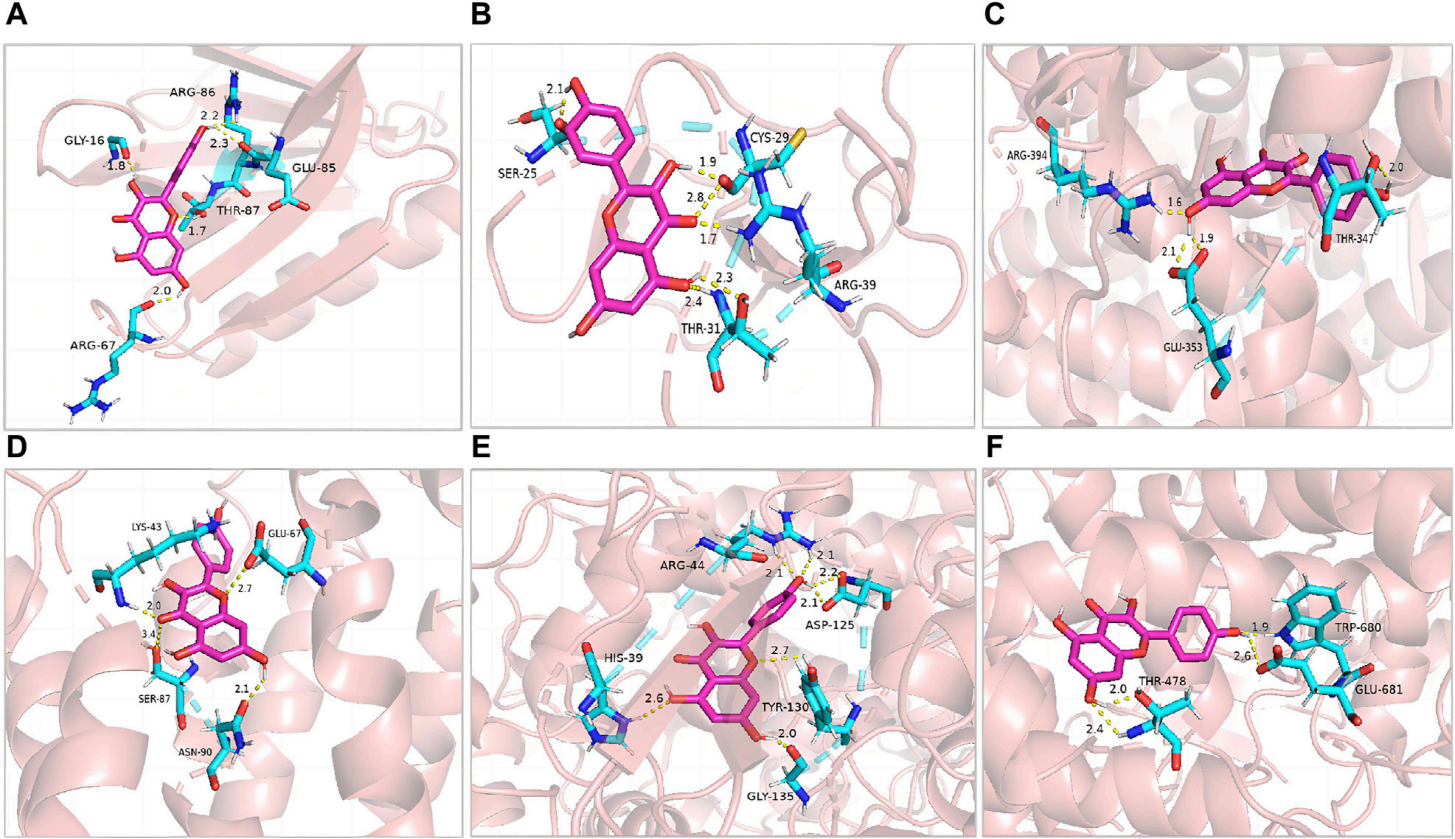
Pharmacological targets of kaempferol in vitiligo. Molecular docking revealed the binding of kaempferol to AKT1 **(A)**, MMP2 **(B)**, ESR1 **(C)**, IL2 **(D)**, PTGS2 **(E)** and MPO **(F)**.

Kaempferol (d = 101) was identified as one of the compounds with the highest degree of association with the constructed networks, according to the network pharmacology analysis, with OB and DL values of 41.88% and 0.24, respectively. Furthermore, in this study, we used detected a kaempferol content of 62.96 ng per 100 g of *V. anthelmintica* (L.) Willd.seed ethanolic extract by MRM approach. However, the specific mechanism of action remains unclear. Next, we conducted molecular docking simulations to explore the binding interactions between kaempferol and five target proteins (AKT1, VEGFA, ESR1, PTGS2, and IL2) that exhibited the highest degree of association with the established networks (Figure 3).

The molecular docking analysis used in this study simulated the binding of kaempferol to the potential key targets, AKT1, MMP2, ESR1, IL2, PTGS2, and MPO. Diagrams of the ligand and potential target docking results are shown in Figure 3. Overall, kaempferol was able to bind to the ARG-86, GLU-85, GLY-16, THR-87, and ARG-67 residues of AKT1 with a binding energy of −6.42 kcal·mol-1. The binding energy of Kaempferol to MMP2 residues, including CYS-29, SER-25, ARG-39, and THR-31, was −5.14 kcal·mol-1.

### 3.6 Kaempferol protects HaCaT cells from AAPH-induced injury

**FIGURE 4:**
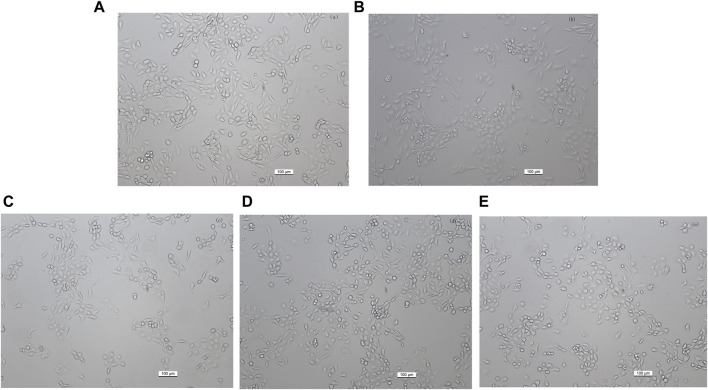
Effect of different kaempferol concentrations on the morphology of HaCaT cells (magnification, ×100). **(A)** Control, untreated HaCaT cells. **(B)** HaCaT cells treated with 25 mM AAPH for 24 h **(C–E)** HaCaT cells pretreated with **(C)** 10 μM, **(D)** 20 µM or **(E)** 30 µM kaempferol for 24 h, followed by incubation with 25 mM AAPH for 24 h.

**FIGURE 5:**
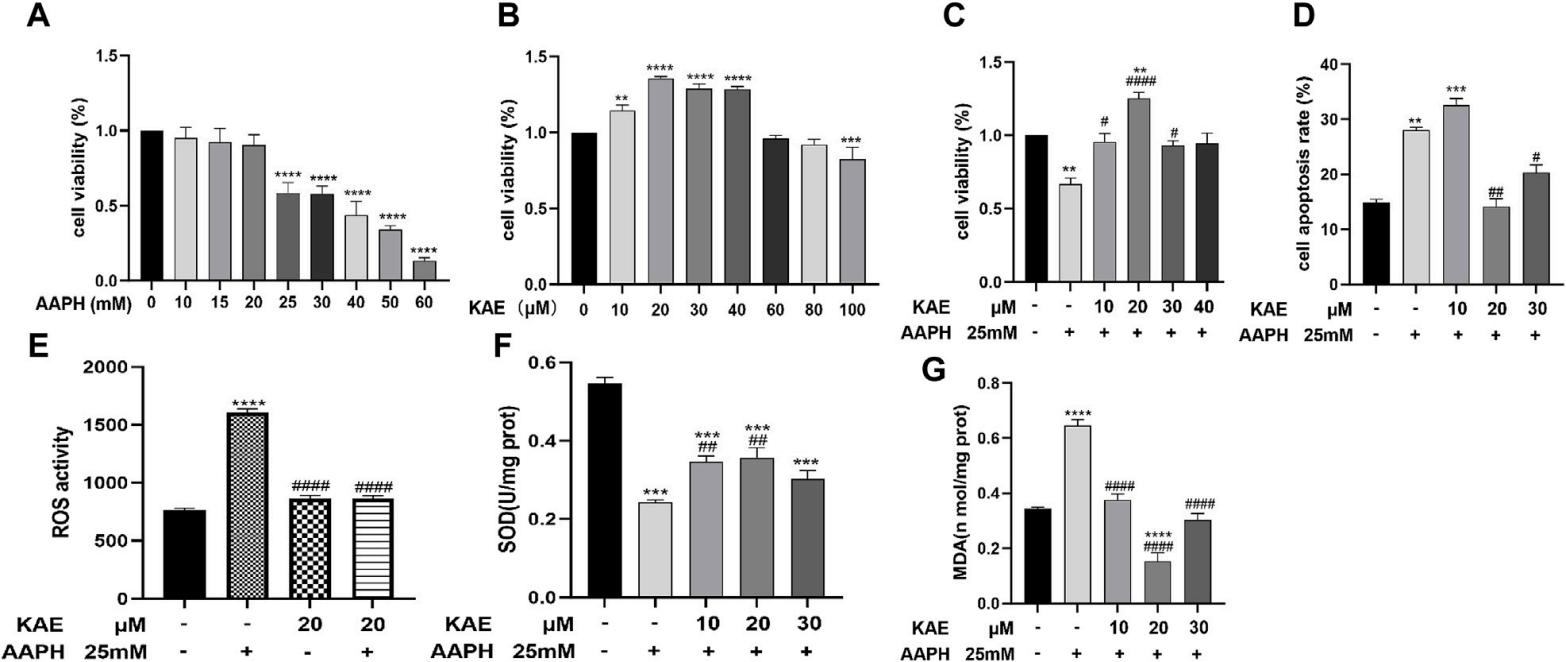
Effect of kaempferol treatment on the viability and apoptotic rate of HaCaT cells under AAPH-induced cytotoxicity. **(A)** HaCaT cells treated with AAPH (0–60 mM) for 24 h **(B)** HaCaT cells treated with kaempferol (0–100 µM) for 24 h **(C)** HaCaT cells pretreated with kaempferol (10, 20, 30, or 40 µM) for 24 h, followed by incubation with 25 mM AAPH for 24 h. **(D)** Apoptotic rate of HaCaT cells treated with AAPH assessed by PI staining and flow cytometry. **(E)** ROS activity, **(F)** SOD activity, and **(G)** MDA content in HaCaT cells. Control, untreated HaCaT cells; AAPH, cells treated with 25 mM AAPH; in the experimental groups, cells were treated with 10, 20, or 30 µM kaempferol for 24 h. The values presented indicate the mean ± SEM of three independent experiments. Effects of kaempferol on AAPH-induced **p < 0.01,***p < 0.001, ****p < 0.0001 *versus* control; ^#^ *p* < 0.05, ^##^ *p* < 0.01, ^####^ *p* < 0.0001 *versus* AAPH-treated cells. AAPH, 2,2-azobis (2-methylpropionamidine) dihydrochloride; KAE, kaempferol; SEM, standard error of the mean.

To investigate the role of kaempferol in the treatment of vitiligo, we constructed a vitiligo oxidative stress model. We first treated HaCaT cells with different concentrations of AAPH for 24 h *in vitro*. In terms of cell morphology, cells in the oxidative stress model group compare with control group displayed slightly elliptical, oval, or round morphology, with blurred edges and reduced adherence (Figure 4B). Nonetheless, after intervention with kaempferol at different concentrations (10, 20, and 30 mM), there was partial restoration of cell morphology (Figures 4C–E). The cell viability results revealed a concentration-dependent reduction in cell viability as the concentration of AAPH increased (Figure 5A). Moreover, cell viability in the AAPH-alone group (>25 mM) was significantly lower than that of the control group (no AAPH treatment), indicating that AAPH induces cytotoxic damage in HaCaT cells (*p* < 0.001). Additionally, the half-maximal inhibitory concentration (IC_50_) value of AAPH was determined to be approximately 25 mM. Therefore, 25 mM AAPH was used in future experiments to establish a HaCaT cell oxidative stress model. Subsequently, we confirmed that the concentration of kaempferol treatment can effect HaCaT cell viability (Figure 5B).

The CCK-8 results revealed the application of kaempferol at concentrations ranging from 10 to 30 µM led to a significant increase in cell viability within AAPH-treated cells, without any apparent cytotoxic effects; in particular, treatment with 20 µM kaempferol exhibit the highest increase in cell viability and provide the highest cytoprotective effect (Figure 5C).

To investigate the effects of kaempferol pretreatment on AAPH-induced apoptosis in HaCaT cells, we conducted propidium iodide (PI) staining (Figure 5D). The corresponding results indicated that the apoptosis rates for the control group, AAPH-alone group, AAPH with 10 μM, 20 µM kaempferol and 30 µM Kaempferol were 14.87% ± 0.35%, 28.03% ± 0.24%, 32.53% ± 0.42%, 14.47% ± 0.42% and 20.37% ± 0.32%, respectively. Thus, when compared to the AAPH-alone group, pre-treatment with 20 µM kaempferol yielded the most significant reduction in apoptosis.

### 3.7 Kaempferol affects AAPH-Induced intracellular SOD, MDA, and ROS activity in HaCaT cells

Next, we evaluated the effects of different concentrations of AAPH on MDA content, SOD enzyme activity, and ROS activity in HaCaT cells. In comparison to the control group, ROS activity (Figure 5E) and SOD level (Figure 5F) were significantly reduced, while MDA content (Figure 5G) was significant increased in HaCaT Cells that were exposed to 25 mM AAPH for 24 h. Nonetheless, pretreatment with 10, 20, or 30 µM kaempferol led to a significant reduction in the elevated MDA content and ROS activity, while increasing the decreased SOD level induced by AAPH. These findings suggest that kaempferol may ameliorate the oxidative and antioxidant imbalances induced by AAPH through its effects on MDA, SOD, and ROS in HaCaT cells.

**FIGURE 6:**
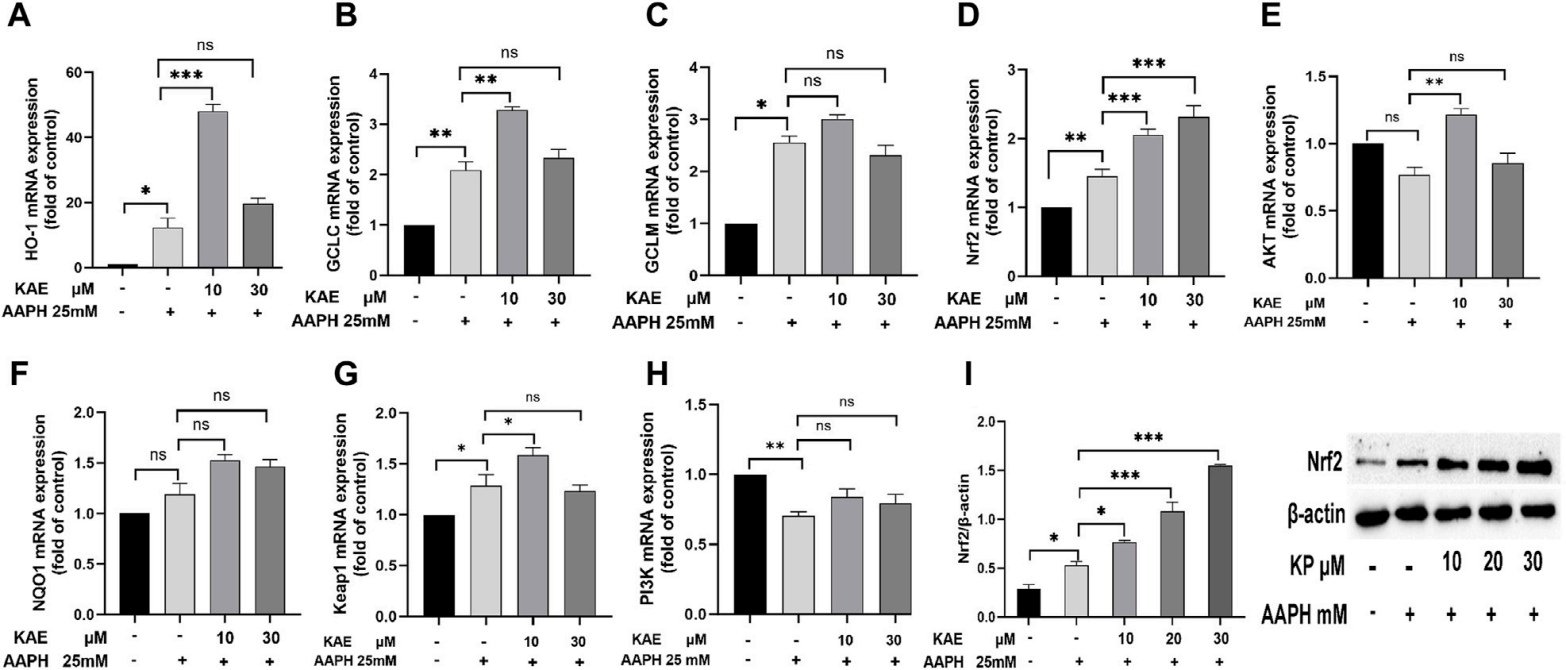
Effects of kaempferol on the expression of **(A)** HO-1, **(B)** GCLC, **(C)** GCLM, **(D)** Nrf2, **(E)** AKT, **(F)** NQO1, **(G)** Keap1 and **(H)** PI3K. **(I)** Effect of kaempferol on the expression of Nrf2 protein in HaCaT cells. AAPH, cells treated with 25 mM AAPH; in the experimental groups, cells were treated with 10 or 30 µM kaempferol for 24 h**p < 0.01,***p < 0.001, ****p < 0.0001 *versus* control. AAPH, 2,2-azobis (2-methylpropionamidine) dihydrochloride; KAE, kaempferol; SEM, standard error of the mean.

Finally, we evaluated the effects of kaempferol on the expression of HO-1, GCLC, GCLM, Nrf2, AKT, NQO1, Keap1 and PI3K. Compared with the control group, 25 mM AAPH can increase the cellular expression levels of HO-1, GCLC, GCLM, Nrf2, NQO1 and Keap1 mRNA, and decrease AKT and PI3K mRNA (Figure 6). Low doses of kaempferol increased Nrf 2, HO-1 and GCLC mRNA expression levels in HaCaT cells, and high doses of KP only increased the intracellular Nrf2 mRNA expression levels. Nrf two protein expression in HaCaT compared with the control group. Different doses of KP can increase the intracellular Nrf two protein expression in a concentration-dependent manner as compared to the model group (Figure 6I).

## 4 Discussion

Vitiligo is a common, acquired, and progressive disorder, characterized by depigmentation of the skin. The complex pathogenesis of vitiligo, oxidative stress injury is a key factor involved in the autoimmune response of vitiligo (Li et al., 2017). Therefore, reducing the level of oxidative stress is an important topic in vitiligo treatment. In Xinjiang, *V. anthelmintica* (L.) Willd has been commonly used to increase melanin production (Zhou et al., 2012), flavonoids and caffeic acid compounds have been identified as some of the most effective components for vitiligo treatment (Tuerxuntayi et al., 2014). Flavonoids, in particular, have received increasing attention due to their antioxidant and anti-inflammatory properties (Panche et al., 2016). Evidence suggests that the flavonoid active compounds have been isolated and identified, such as isorhamnetin, Butin and luteolin. These flavonoids can reduce ROS accumulation and promote melanogenesis (Lai et al., 2021; Hu et al., 2023). However, the current anti-vitiligo treatment for *Vernonia anthelmintica* (L.) Willd. is in preliminary stages, exact therapeutic effect and the treatment targets of the active ingredient have not yet been established.

To the best of our knowledge, this is the first comprehensive study that combines SRM/MRM approaches with network analysis to explore the active flavonoid compounds within *V. anthelmintica* (L.) Willd. Overall, our study highlights the protective effects of the active ingredient of *V. anthelmintica* (L.) Willd., kaempferol, on keratinocytes in mitigating AAPH-induced damage. In the present study, we identified 36 flavonoids within *V. anthelmintica* (L.)Willd seeds. Furthermore, by employing network pharmacology, we identified bioactive compounds and conducted ADME screening of these 36 isolated flavonoids, which ultimately revealed the presence of 14 compounds with druggability, including kaempferol, kaempferide, and eriodictyol. In total, we identified 1286 vitiligo-related genes and 211 target genes associated with the 14 identified compounds, which were used to, ultimately, identify a total of 43 component–vitiligo common targets.

Here, we employed GO analysis to investigate the 43 previously identified common targets of *V. anthelmintica* (L.) Willd. that are associated with to vitiligo. These critical targets were mainly involved in processes related to oxidative stress, inflammation, cell growth, metabolism and immunity. Notably, biological processes such as the regulation of inorganic substances, mitochondrial membrane potential, and mitochondrial organization were significantly enriched. Mitochondria are not only recognized as the source of ROS; they are also the primary organelles susceptible to the harmful effects of oxidative stress. Any impairment of mitochondrial function can subsequently affect melanocyte survival (Zhou et al., 2018). Increasing evidence has demonstrated that mitochondria are crucial in oxidative stress–mediated melanocyte apoptosis, highlighting mitochondria as potential targets for clinical vitiligo therapy (Wang et al., 2019b; Jiang et al., 2019).

KEGG pathway analysis further emphasized the relationship between these 43 common targets and vitiligo. Corresponding significantly enriched pathways included the PI3K-AKT signaling pathway, chemical carcinogenesis receptor activation, the estrogen signaling pathway, endocrine resistance, and the C-type lectin receptor signaling pathway. Notably, dysregulation in the PI3K/AKT signaling pathway has been implicated in various diseases associated with melanocytes, including melanoma, chloasma, and vitiligo. In melanocytic hyperproliferative diseases such as melanoma, the PI3K/AKT signaling pathway is overactivated, which promotes cell proliferation and inhibits apoptosis, facilitating tumor cell metastasis (Zhu et al., 2018). However, under H_2_O_2_-induced oxidative stress, the PI3K/AKT signaling pathway in melanocytes is inhibited, leading to increased apoptosis (Yang et al., 2016; Zhou et al., 2016). Activation of the PI3K-Akt signaling pathway protects against oxidative stress-induced cell death, often associated with the pathogenesis of vitiligo (Yang et al., 2016). The PI3K/Akt pathway can be activated by the basic fibroblast growth factor (bFGF) to increase cell proliferation, migration, and differentiation of several types of cells including melanocytes (Shi et al., 2016). This suggests that the identified compounds might also exert beneficial effects on vitiligo by activated PI3K/AKT signaling pathway. In summary, these findings indicate that 43 common targets identified in *V. anthelmintica* (L.) Willd. may be involved in vitiligo-related pathways, highlighting the potential of *V. anthelmintica* (L.) Willd. in the treatment of vitiligo. Thus, further study into these genes and pathways is certainly warranted.

The five key target genes (*AKT1*, *VEGFA*, *ESR1*, *IL2*, and *MPO*) identified by the compound–target network were closely related to the pathogenesis and/or treatment of vitiligo. In the present study, molecular docking simulations revealed that kaempferol exhibits high binding affinities with the five key target genes. AKT1 induces mitochondrial ROS production and mitophagy in macrophages; subsequently, AKT1-mediated mitophagy can then increase resistance to alveolar macrophage apoptosis and pulmonary fibrosis (Larson-Casey et al., 2016). Kaempferol, a flavonoid compound derived from plants, is known for its various functions, including antioxidant, anti-cancer, anti-inflammatory, and anti-apoptotic properties (Fouzder et al., 2021). Moreover, it was found to be one of the compounds with the highest degree of association (d = 101) in our network pharmacology analysis. While current literature on the direct impact of Kaempferol on mitochondrial function is limited, the implication of interaction with AKT1 indicates a potential pathway where Kaempferol could improve vitiligo by modulating mitochondrial and cell apoptosis. Further investigation is required to elucidate this mechanism and its clinical relevance. ESR1 has been reported as one of the proteins contributing to vitiligo susceptibility. Notably, this protein may be an important target for the successful treatment of vitiligo using a steroid–thyroid hormone mixture containing estrogen (Malhotra et al., 2018). VEGFA, also known as vascular endothelial growth factor A, is considered to be a particularly interesting candidate gene, which has been attributed to its polymorphisms, its correlation with autoimmune disorders, and its induction of angiogenic factor expression in vitiligo; moreover, VEGFA may be a potential target for late-onset vitiligo therapy (Almasi-Nasrabadi et al., 2019). IL2 has been implicated in the regulatory pathways of autoimmune diseases, including signaling and promotion of the growth and proliferation of T cells; therefore, this protein plays an important role in generating immune responses (Martins et al., 2020). Finally, MPO-specific markers are strongly associated with microscopic polyangiitis and other autoimmune diseases (Prendecki et al., 2021). Given that VEGFA, IL2, and MPO all play roles in autoimmune diseases, in this study, kaempferol has been shown to bind well with these molecules, which suggests a potential avenue for therapeutic interventions targeted at these molecular interactions in the context of vitiligo. Overall, numerous studies have indicated the association between these key target proteins and vitiligo, supporting the understanding that *V. anthelmintica* (L.) Willd. possesses antioxidant properties. Similarly, we demonstrated that kaempferol has good binding activity with these critical target proteins, including AKT1, ESR1, and PTGS2 (Jiang et al., 2022). Ultimately, these findings support the potential role of kaempferol as an active ingredient in *V. anthelmintica* (L.) Willd. for the treatment of vitiligo.

To further demonstrate the potential therapeutic effects of kaempferol on vitiligo, we performed *in vitro* cellular experiments. In these experiments, HaCaT cells were pretreated with kaempferol or left untreated before stimulation with AAPH to induce oxidative damage. Overall, pretreatment with kaempferol increased HaCaT cell viability, reduced apoptosis, lowered MDA content and ROS activity, and enhanced SOD activity. And kaempferol was also found to increase the expression of cellular antioxidant genes, including HO-1, GCLC, GCLM, Nrf2, NQO1 and Keap1. These findings indicate that kaempferol has the capacity to mitigate oxidative damage, offering promising prospects for its use as an antioxidant therapy for the treatment of vitiligo.

Despite the insights gained from network pharmacology, molecular docking, and *in vitro* experiments in identifying potential compounds and targets of *V. anthelmintica* (L.) Willd. in the context of vitiligo, this study has few limitations. First, the lack of *in vivo* analysis and limited *in vitro* experiments restricts the directly validation of the antioxidative effects and associated pathways of the identified compounds–target pairs. Furthermore, while the protective effects of kaempferol against AAPH-induced oxidative damage in HaCaT cells was investigated, further experiments and analysis are warranted to ascertain the exact impact of kaempferol on AAPH-induced melanocytes. Such investigations would, ultimately, facilitate the development of kaempferol as an antioxidant source and offer a more robust theoretical basis for targeted vitiligo therapy.

## 5 Conclusion

In conclusion, our research represents the first to comprehensive analysis of active flavonoid compounds in *V. anthelmintica* (L.) Willd via the combination of SRM/MRM and network analysis approaches*.* These results revealed the protective effects of its active ingredient, kaempferol, against AAPH-induced damage in keratinocytes. Moreover, compound-target network analysis identified 14 key compounds of *V. anthelmintica* (L.) Willd., including kaempferol, kaempferide, and eriodictyol; notably, these compounds exhibit significant druggability, with the potential to be used for the treatment of vitiligo. Further, we identified *AKT1*, *VEGFA*, *ESR1*, *PTGS2*, and *IL2* as key therapeutic target genes, which possessed anti-oxidative properties. Among the significant pathway identified, the PI3K/AKT signaling pathway was of particular note. Moreover, kaempferol was identified as one of the key compounds in *V. anthelmintica* (L.) Willd. that exhibited a high degree of binding with these critical targets. *In vitro* cell experiments also showed that kaempferol increased HaCaT cell viability, reduced apoptosis, lowered MDA content and ROS activity, and enhanced SOD activity, effectively providing resistance against oxidative stress damage. In summary, this study highlights the promising potential of kaempferol as an antioxidant treatment for vitiligo.

## Data Availability

The original contributions presented in the study are included in the article/Supplementary material, further inquiries can be directed to the corresponding author.
